# Morphological Changes and Strong Cytotoxicity in *Yarrowia lipolytica* by Overexpressing Delta-12-Desaturase

**DOI:** 10.3390/jof10020126

**Published:** 2024-02-03

**Authors:** Yufei Chang, Zhen Wang, Hequn Li, Wenrui Dang, Yuanda Song, Xinxin Kang, Huaiyuan Zhang

**Affiliations:** 1 Colin Ratledge Center for Microbial Lipids, School of Agricultural Engineering and Food Science, Shandong University of Technology, Zibo 255049, China19811715276@163.com (H.L.);; 2 School of Public Health, Qilu Medical University, Zibo 255300, China

**Keywords:** lipid, oxidative stress, delta-12 desaturase, linoleic acid, *Yarrowia lipolytica*

## Abstract

In this study, delta-12 desaturase was overexpressed in *Yarrowia lipolytica* using the single-copy integrative vector pINA1312 and multicopy integrative vector pINA1292, resulting in the engineered yeast strains 1312-12 and 1292-12, respectively. The content of intracellular linoleic acid (LA) in the 1292-12 strain was much higher than in the 1312-12 strain and the control group. One interesting finding was that the 1292-12 strain showed obvious changes in surface morphology. The 1292-12 colonies were much smaller and smoother, whereas their single cells became much larger compared to the control strain. In addition, the dry cell weight (DCW) of the 1292-12 strain was obviously increased from 8.5 to 12.7 g/L, but the viable cell number sharply decreased from 10^7^ to 10^5^/mL. These results indicated that increased LA content in *Yarrowia lipolytica* could induce morphological changes or even oxidative stress-dependent cell death. The reactive oxygen species (ROS) and malondialdehyde (MDA) were accumulated in the 1292-12 strain, while the antioxidant activities of intracellular catalase (CAT) and superoxide dismutase (SOD) were significantly decreased by 27.6 and 32.0%, respectively. Furthermore, it was also revealed that these issues could be ameliorated by the exogenous supplementation of vitamin C, fish and colza oil.

## 1. Introduction

In addition to being a rich source of energy, similar to carbohydrates, polyunsaturated fatty acids (PUFAs) are also required for the maintenance of membrane structure, cell growth and proliferation [1]. They are regarded as the key compounds in maintaining the physiological functions of cell membranes and the adaption to variable environments, which is determined by the number of double bonds in the hydrocarbon chain of the PUFAs. Furthermore, they are also the precursors of important mediators involved in a number of biochemical reactions in living organisms.

However, these compounds are also toxic to eukaryotic cells, and may cause apoptosis under specific conditions [2]. Several previous experimental studies have shown that certain PUFAs have inhibitory effects on the growth of tumor cells both in vitro and in vivo [3,4,5]. These fatty acids, such as linoleic acid (LA, C18:2), α-linolenic acid (ALA, C18:3) and arachidonic acid (ARA, C20:4) may trigger the apoptotic death of human smooth muscle cells at certain concentrations in the nutrient medium [6]. The cytotoxicity of PUFAs is related to the length of the carbon chain (r = 0.602) and the number of double bonds (r = 0.956) [7]. In addition, the cytotoxic or antiproliferative effects of specific PUFAs vary considerably depending on the experimental cell types and culture conditions. Their effects are complex and may be associated with changes in the membrane composition, oxidative stress and metabolism, resulting in the generation of active compounds [8]. Therefore, studying the influence of PUFAs on different cell types is necessary for understanding their antiproliferative activities and modulatory roles relevant to cell growth.

Several studies have shown that ω-6 PUFAs, such as LA, play a prominent role in apoptosis in vivo [9]. The addition of exogenous LA can cause cell death in *Saccharomyces cerevisiae* by aggravating the oxidative stress response under certain conditions [10]. However, there are few studies on the cytotoxicity of endogenous LA, and the mechanism of its toxic effects in eukaryotic cells remains elusive. The major fatty acid species in *Yarrowia lipolytica* are saturated or monounsaturated fatty acids with 16 or 18 carbon atoms, and LA accounts for a low proportion of total fatty acids. In this study, delta-12 desaturase was chromosomally overexpressed in *Yarrowia lipolytica* and the resulting engineered strain was characterized in detail. Subsequently, the effects of endogenous LA on cell viability and morphology were examined and the intracellular levels of ROS, MDA, CAT and SOD were measured to further elucidate the molecular mechanism of PUFA-induced apoptosis. Furthermore, the supplementation of vitamin C and the addition of fish or colza oil to the medium were found to reduce oxidative stress and have positive effects on mitigating the morphological changes induced by excess LA.

## 2. Materials and Methods

### 2.1. Strains and Culture Conditions

The *Y. lipolytica* Polh (URA3^−^) used in this study was derived from the wild-type strain ATCC 20460. *Escherichia coli* Top10 was used for plasmid construction. Endogenous delta-12 desaturase gene was cloned from genomic DNA into the plasmids pINA1312 (single-copy integration) and pINA1292 (multicopy integration) for protein expression, and inserted into the yeast genome according to a previously described method [11].

The recombinant yeast strains were cultivated in YPD medium (10 g/L Bacto-yeast extract, 20 g/L Bacto-peptone, 20 g/L glucose) for 48 h, and then transferred into the fermentation medium (1.5 g/L yeast nitrogen base without amino acids and ammonium sulphate, 2.0 g/L ammonium sulfate, 1 g/L yeast extract, 90 g/L glucose) at a 2% inoculation rate for fermentation. The strains were cultivated in 300 mL fermentation medium in 1000 mL baffled shake flasks for 144 h at 28 °C with agitation at 200 rpm [11].

### 2.2. Analytical Methods

The yeast cells were collected at 24 h intervals for observation of cell morphology by using an optical microscope (OM) and scanning electron microscope (SEM) [11]. The samples were separated by centrifugation, and the supernatant was collected for determination of residual glucose and NH_4_^+^ using respective kits [11,12,13]. The cell pellet was washed twice with distilled water to measure the DCW and for fatty acid analysis. The washed yeast cells were also broken by vigorous vortexing with glass beads and separated by centrifugation at 3000× *g* for 15 min at 4 °C, the cells supernatant was used for MDA, SOD and CAT measurement, ROS was determined by using live cells [9].

### 2.3. Construction of the 1312-12 and 1292-12 Strains

The strains 1312-12 and 1292-12 were constructed based on the pINA1312 (single-copy integration) and pINA1292 (multicopy integration) plasmids, using a 1.2 kb designed rDNA fragment from *Y. lipolytica* to replace the original URA3 gene for targeted homologous recombination. Vectors, pINA1312 and pINA1292 with non-defective URA3 allele (*ura3d1*) and defective URA3 allele (*ura3d4*) were stored in our laboratory. The restriction endonucleases *Pml* I and *Kpn* I were used to generate pINA1312-Δ12Des and pINA1292-Δ12Des by synonymous change of the nucleotides using appropriate primers. All constructed vectors were screened by enzyme digestion and PCR, and then confirmed by DNA sequencing.

### 2.4. Lipid Extraction and Gas Chromatography for Fatty Acid Analysis

The harvested yeast cells were washed twice with distilled water and then freeze-dried for biomass and lipid analysis. The total fatty acid (TFA) was extracted based on a previously described method. Yeast cells were lysed with hydrochloric acid and TFA was extracted into chloroform: methanol (2:1, *v*/*v*) by adding pentadecanoic acid (C15:0) as an internal standard. After methyl esterification by 10% (*w*/*w*) hydrochloric methanol, the fatty acid methyl ester was determined by gas chromatography (7890B, Agilent, Santa Clara, CA, USA) with an Agilent 123–3232 column (30 m × 320 μm × 0.25 μm). The temperature gradient was as follows: initial temperature 80 °C for 3 min, increasing to 160 °C at 10 °C/min, then to 230 °C at 5 °C/min, and holding for 2 min [11,13].

### 2.5. Cell Viability Assay

The cell viability was determined by a spot test on YPD plates [14]. Yeast cells from the exponential phase of fermentation were harvested by centrifugation and resuspended in sterile saline [11]. After 10-fold dilution, 100 μL of each dilution were spotted on the YPD plates and incubated 2–3 days at 28 °C. To count the number of viable cells with high reliability, three replicates of each cell suspension were quantified, and the average cell number of each sample was calculated.

### 2.6. Supplementation of Vitamin C

Vitamin C was the key factor for many biological reactions and was well known to promote cell growth. Some efficacy studies of its antioxidant activity suggested that supplementation of vitamin C could be an alternative method to block iron-induced oxidative damage. In order to explore the effect and mechanism of the cytotoxicity of LA in *Y. lipolytica*, vitamin C was added to the fermentation medium at the starting point to a final concentration of 250 mg/L [14].

### 2.7. Detection of ROS and MDA

The ROS detection assay kit (CA1410, Sulaibio, Beijing, China) is designed for the detection of reactive oxygen species in live cells, and it is one of the most commonly used methods based on the change of fluorescence intensity of 2,7-dichlorofluorescin diacetate (DCFH-DA), which is easily oxidized into fluorescent dichlorofluorescein (DCF) by ROS. The cells were harvested by centrifugation (6000× *g*, 5 min) and resuspended in 10 μM DCFH-DA for 30 min at 37 °C in the dark. Subsequently, they were centrifuged and resuspended in 40 μL PBS buffer, followed by fluorescence microscopy using an excitation wavelength of 500 nm and an emission wavelength of 525 nm [15].

The MDA concentration was determined using the MDA assay detection kit (BC0025, Sulaibio, Beijing, China) based on thiobarbituric acid, and the procedures were carried out in accordance with the kit instructions. The reaction assays were incubated in a water bath at 95 °C for 40 min, after which the absorption of the MDA reaction product was measured at a wavelength of 532 nm [15].

### 2.8. CAT and SOD Enzyme Activity Assays

The enzyme activity of CAT was quantified via the degradation of H_2_O_2_ using a CAT detection kit (BC0205, Sulaibio, Beijing, China). The specific activity was expressed as 1 mM H_2_O_2_ reduced per min and mg protein. Cell supernatants were mixed with the assay buffer, after which the remaining H_2_O_2_ concentration was measured by UV spectrophotometry at a wavelength of 240 nm.

The activity of SOD was determined using an SOD detection kit (BC5165, Sulaibio, Beijing, China) on the basis of inhibition of the superoxide-dependent reduction of hydroxylamine by xanthine oxidase [16]. The amount of SOD required to inhibit the rate of hydroxylamine reduction by 50% was defined as 1 unit of activity. The enzyme reaction assays were incubated for 30 min at 37 °C, and then the hydroxylamine reduction was measured by UV spectrophotometry at a wavelength of 560 nm.

### 2.9. Supplementation of Fish and Colza Oil

In order to alleviate the toxicity of LA in *Y. lipolytica*, fish and colza oil were supplied in the fermentation medium at a final concentration of 10 g/L. The fish and colza oil were emulsified using the surfactants Tween 80 (Sinopharm, Beijing, China) at a final concentration of 100 mg/L with only Tween 80 added in the control culture, and then sterilized by filtration through a 0.22 μm pore-size membrane.

### 2.10. Statistical Analysis

Statistical analysis was carried out using GraphPad Prism 5.0 Software (La Jolla, CA, USA). Unless otherwise indicated, all data were presented as means ± standard deviations (SD). Unpaired Student’s *t*-test was used to assess the statistical significance of differences between two groups. Differences with *p*-values lower than 0.05 were considered statistically significant.

## 3. Results

### 3.1. Influence of Delta-12 Desaturase Expression on the Fatty Acid Profile

To obtain a yeast strain with high intracellular LA content, delta-12 desaturase was overexpressed in *Y. lipolytica* using the integrative plasmids pINA1312 and pINA1292, resulting in the mutant strains 1312-12 and 1292-12, as well as two control strains with the empty plasmids. The mutants were grown in a medium with a high C/N molar ratio of 80 to promote the synthesis of fatty acids. The effect of delta-12 desaturase expression on fatty acid synthesis in *Y. lipolytica* was analyzed by GC. The fatty acid profile of the yeast cells mainly included palmitic acid (C16:0), palmitoleic acid (C16:1), stearic acid (C18:0), oleic acid (C18:1) and linoleic acid (C18:2). Furthermore, the overexpression of delta-12 desaturase had a significant influence on the fatty acid profile of the engineered strains, especially C18:2 and C18:1 (Table 1). The strain 1292-12 with multicopy integration of the delta-12 desaturase gene accumulated much more C18:2 than the single-copy strain 1312-12 and the control strain. In fact, the C18:2 content of 1292-12 reached up to 30.8% of TFA, while it was only 14.6 and 8.7% in the single-copy strain 1312-12 and the control strain, respectively. However, there was no significant difference in the TFA content (of their DCW) between the transformants and control strain (Table 1).

### 3.2. Influence of Delta-12 Desaturase Overexpression on the Growth of Y. lipolytica

**Figure 1:**
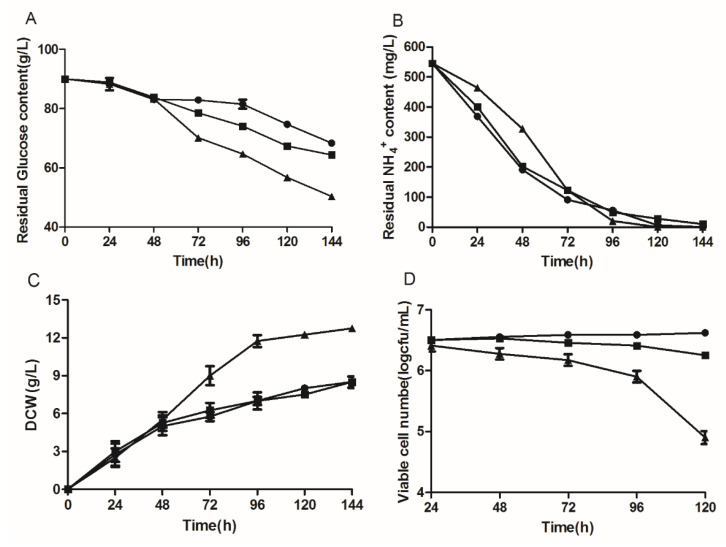
The residual glucose concentration (**A**), residual NH_4_^+^ concentration (**B**), dry cell weight (**C**) and viable cell number (**D**) in engineered strains of *Y. lipolytica* with different expression levels of delta-12 desaturase. The control strain, circles (●); Single-copy strain 1312-12, squares (■); Multicopy strain 1292-12, triangles (▲).

To assess the effect of delta-12 desaturase overexpression on growth, the engineered strains were cultivated in fermentation medium, and the consumption of glucose and NH_4_^+^, as well as the DCW and cell viability were determined (Figure 1). The analysis results showed that the time-course of NH_4_^+^ consumption was similar between the control and engineered strains (1312-12 and 1292-12). However, the total consumption of glucose was obviously higher in 1292-12 than in the control strain. At the same time, the DCW of 1312-12 and the control strain reached 8.5 g/L during the logarithmic phase, exhibiting a similar growth curve. By contrast, the highest DCW of strain 1292-12 with the multicopy integration vector increased by 50% and reached approximately 12.7 g/L after 144 h of cultivation (Figure 1C). However, the viable cell number of strain 1292-12 sharply decreased from 10^6^ to 10^4^/mL, while there was only a modest decrease in the 1312-12 strain when compared with the control strain (Figure 1D). To sum up, these results indicated that the morphology of the engineered *Y. lipolytica* was larger, but the number of living cells significantly decreased due to the multicopy overexpression of the delta-12 desaturase gene.

### 3.3. Effects of Linoleic Acid Toxicity on the Cell Morphology of Y. lipolytica

**Figure 2:**
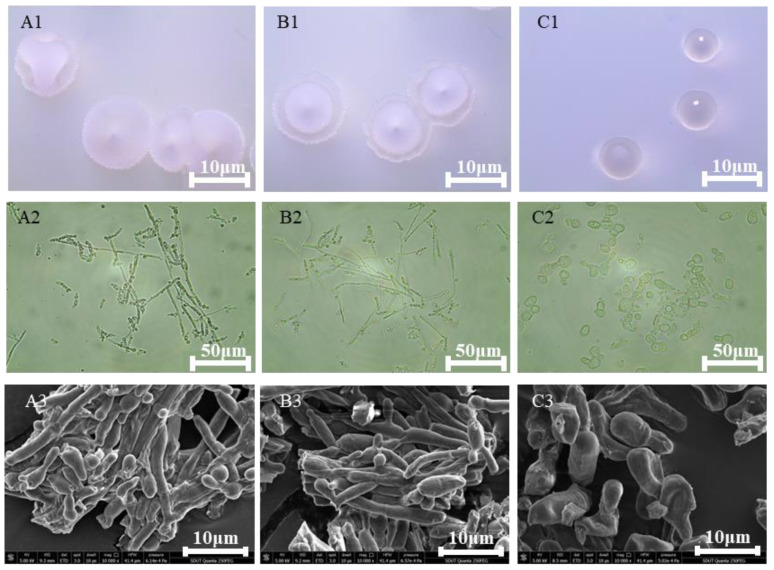
The influence of delta-12 desaturase overexpression on the colony (**A1**–**C1**) and cell morphology (**A2**–**C2**,**A3**–**C3**, 10,000× magnification) of *Y. lipolytica*. (**A1**–**A3**) the colony and cell morphology of the control strain; (**B1**–**B3**) the colony and cell morphology of the single-copy strain 1312-12; (**C1**–**C3**) the colony and cell morphology of the multicopy strain 1292-12.

The overexpression of the delta-12 desaturase gene in *Y. lipolytica* resulted in dramatic changes of colony and cell morphology visible macroscopically, under an optical microscope, as well as SEM. Morphological analysis indicated that the colony surface and edges of the control strain were rough, with obvious wrinkles (Figure 2(A1–A3)). By contrast, the wrinkles at the edges of the colonies of 1312-12 were greatly reduced, and a transparent circle formed around the colony (Figure 2(B1–B3)). Moreover, the multicopy overexpression strain 1292-12 exhibited even more pronounced changes. The wrinkles completely disappeared, and the border of the colony was smooth and transparent (Figure 2(C1–C3)). Previous studies showed that *Y. lipolytica* cells gradually formed a pseudomycelium from single cells during the process of fermentation [11]. In our study, a mixture of single yeast cells and short mycelial chains was observed in the control and single-copy strain 1312-12. The SEM results revealed that the cell surface of the 1312-12 strain was relatively smooth, and there was no significant change compared with the control strain (Figure 2(B1–B3)). However, the cell morphology of the 1292-12 strain was completely changed, and the strain formed bigger single cells, which were indented and rough on the surface due to the lipotoxicity of high LA accumulation (Figure 2(C1–C3)).

### 3.4. The Lipotoxicity of Excess LA in the Multicopy Strain 1291-12 Is Associated with Oxidative Stress

**Figure 3:**
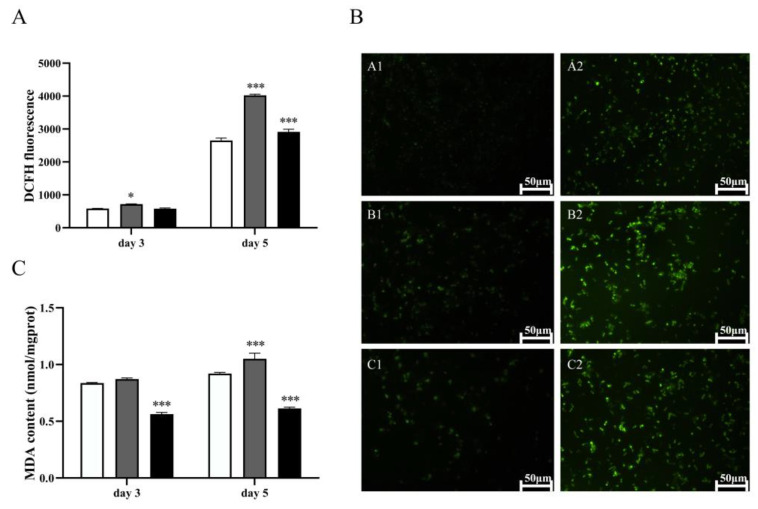
ROS and MDA content of the control and multicopy strain 1292-12. (**A**) the accumulation of ROS measured by DCFH fluorescence at 72 h and 120 h. (**B**) fluorescence image of ROS on days 3 and 5. The control strain (**A1**,**A2**); Strain 1292-12 (**B1**,**B2**); Strain 1292-12 treated with vitamin C (**C1**,**C2**). (**C**) the accumulation of MDA at 72 h and 120 h. The control strain (white column); Strain 1292-12 (gray column); Strain 1292-12 treated with vitamin C (black column). * indicates *p*-values < 0.05, *** indicates *p*-values < 0.001.

To determine if the observed high lipotoxicity of LA is associated with the induction of oxidative stress, we measured the content of intracellular ROS and MDA in the multicopy strain 1292-12. PUFAs such as LA are among the main target molecules of ROS, which may initiate the process of autocatalytic lipid peroxidation and produce harmful substances such as MDA, resulting in the destruction of cell structure and even cell death. The results of DCFH fluorescence analysis showed that the ROS content of strain 1292-12 was significantly higher than that of the control strain, especially after 5 days of fermentation (Figure 3A). This was confirmed by the changes of average fluorescence intensity in the ROS assay of the 1292-12 and control strains (Figure 3B). The intracellular ROS of these strains could attack the highly accumulated LA and then produce a large amount of MDA. Accordingly, the MDA content of strain 1292-12 was also higher than that of the control strain (Figure 3C), indicating an increase in the lipid peroxidation level. However, it was also found that there was a major decrease in ROS and MDA levels in strain 1292-12 after treatment with the classical antioxidant vitamin C (Figure 3A,C) and there was no change in the control strain, a free radical scavenger that can significantly reduce oxidative stress responses.

**Figure 4:**
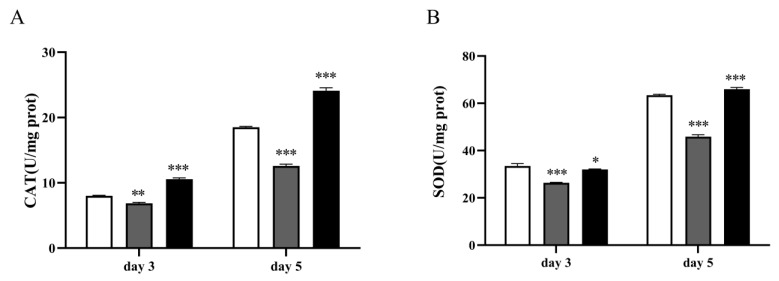
The activities of CAT (**A**) and SOD (**B**) of the 1292-12 and control strains on days 3 and 5. The control strain (white column); Strain 1292-12 (gray column); Strain 1292-12 treated with vitamin C (black column). * indicates *p*-values < 0.05, ** indicates *p*-values < 0.01, *** indicates *p*-values < 0.001.

Previous studies showed that the antioxidant defense systems, such as superoxide dismutase (SOD) and catalase (CAT), were unable to inhibit the production of ROS in the cells under oxidative stress conditions [10]. Thus, the results indicated that the multicopy strain 1292-12 may be more sensitive to oxidative stress due to the high lipid peroxidation levels. To further elaborate this hypothesis, the CAT and SOD activities were measured. As shown in Figure 4, the SOD and CAT activities of strain 1292-12 were lower than that in the control strain after 5 days of fermentation, decreasing by 31.93 and 27.57%, respectively. This indicated that their cells cannot resist the oxidative stress. However, the activities of the detoxifying enzymes in strain 1292-12 increased substantially after treatment with the antioxidant vitamin C (Figure 4), and there was no change in the control strain.

### 3.5. Effect of Fish or Colza Oil Supplementation on the Fatty Acid Profile of Y. lipolytica

As an oleaginous yeast, *Y. lipolytica* is intrinsically capable of producing high levels of microbial lipids through the Kennedy pathway when grown on edible oils as carbon sources [12]. Thus, in order to alleviate the toxic effects of LA on the cellular membranes in *Y. lipolytica*, pre-emulsified fish and colza oil were added to the fermentation medium, respectively. The results confirmed that *Y. lipolytica* could grow well by using these oils as auxiliary carbon sources, and the fatty acid profiles reflected the incorporation of the externally added lipids into the cells (Table 2). The results indicated that the engineered strain 1292-12 still maintained a higher C18:2 content in various culture media than the control strains. However, supplementation of the medium with fish or colza oil caused a significant decrease of the C18:2 content at both 72 and 120 h of fermentation. In addition, the fatty acid profile shifted towards more polyunsaturated fatty acids (C18:3, C20:5, C22:1, C22:6) when either fish oil or colza oil was added to the medium.

### 3.6. Effects of Fish or Colza Oil Supplementation on Cell Growth

**Figure 5:**
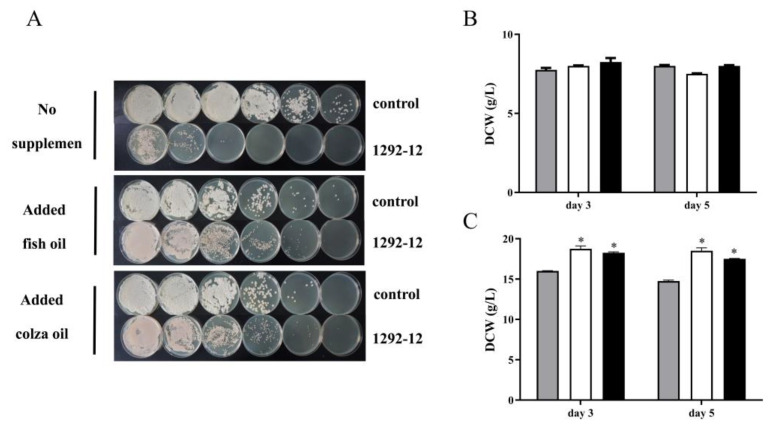
Viability and biomass accumulation of the 1292-12 and control strains in medium supplemented with fish or colza oil. (**A**) The cell viability of the control and 1292-12 strains in medium with fish or colza oil supplementation. (**B**) The dry cell weight of the control strain in non-supplemented medium (gray column), fish oil supplemented medium (white column) and colza oil supplemented medium (black column). (**C**) The dry cell weight of 1292-12 strain in non-supplemented medium (gray column), fish oil supplemented medium (white column) and colza oil supplemented medium (black column). * indicates *p*-values < 0.05.

To investigate the effects of fish or colza oil on cell viability, the 1292-12 and control strains were cultured in fermentation medium and on YPD agar plates, and the number of yeast colonies that grew on the plates was quantified. The results showed that there were less viable cells of strain 1292-12 in non-supplemented medium due to the toxicity of LA compared with the control strain (Figure 5A). There were more colonies on the plates when the strain 1292-12 was grown in a medium with fish or colza oil with the same inoculum size. Thus, adding fish or colza oil to the fermentation medium effectively protected 1292-12 cells from the lipotoxicity of excess LA. Accordingly, while there was no obvious change in the control group (Figure 5B), the DCW of strain 1292-12 increased by nearly 20% when fish or colza oil was added as an auxiliary carbon source (Figure 5C).

## 4. Discussion

PUFAs are not only essential components of cellular membranes and are involved in many biochemical responses in all eukaryotes, but are also the major target of oxidative damage due to lipid peroxidation, which can lead to the generation of cytotoxic aldehydes [17]. Previous studies showed that the addition of extracellular PUFAs reduces cell growth in *S. cerevisiae* at high concentrations [10]. Nevertheless, there are still little data on the toxicity of endogenously synthesized LA, which precludes a real understanding of the underlying mechanisms. Thus, the objective of this study was to investigate the potential effects and mechanisms underlying the toxicity of endogenously produced LA in yeast cells.

Previous studies demonstrated that *S. cerevisiae* could accumulate PUFAs and incorporate them into its cellular membranes, but this resulted in a reduction of the specific growth rate and cell viability due to oxidative stress [10]. In addition, the deletion of FAD12 in *Komagataella phaffii* led to a defect in the synthesis of LA, which resulted in less toxic byproducts of lipid peroxidation and cell death. In this study, delta-12 desaturase was overexpressed in *Y. lipolytica* by single- and multicopy integration to construct the engineered strains 1312-12 and 1292-12, respectively, with different LA contents. Our study results confirmed that the control strain had the lowest LA content, and a high intrinsically produced LA content was clearly a potent inducer of cell death. Cell death usually occurs by one of two distinct mechanisms: apoptosis and necrosis. Apoptosis is a process that can be characterized by dynamic changes of cell morphology, while necrosis can be characterized by early loss of cytomembrane integrity [18,19]. The results of this study indicated that the lipotoxicity of excess LA significantly reduced cell viability (Figure 1), changed the cell morphology (Figure 2), and finally induced cell death. Under oxidative stress conditions, endogenous antioxidant defenses of cells were unable to completely neutralize the free radicals generated by the excessive production of ROS, resulting in severe cell damage and even cell death. In agreement with these observations, PUFA-producing strains were reported to have a shorter lifespan due to an increased amount of ROS [20], which can attack the double bonds of PUFAs, change cytoplasmic membrane fluidity, and induce lipid peroxidation [10,21,22]. The resulting lipid breakdown products include especially harmful cytotoxic aldehydes, which can disrupt cellular metabolism and normal biological functions of the cell [23,24,25]. Our results indicated that total cellular ROS and MDA levels were significantly increased in the multicopy strain 1292-12 (Figure 3), and there was a gradual decrease in CAT and SOD activity during the late stages of fermentation in 1292-12 cells (Figure 4), which diminished the antioxidant capacity of the cells. Therefore, oxidative stress mediated the excessive cell death in strain 1292-12 induced by the high LA content.

Cell membrane fluidity plays an important role in cell survival under the influence of environmental perturbations [2]. Some studies have demonstrated that the toxic effects of LA on the yeast *Y. lipolytica* may be partially linked to a strong interaction of this compound with membrane phospholipids [26,27]. In this model, an increased proportion of PUFAs increases membrane fluidity, while a decreased proportion of PUFAs enhances membrane rigidity. Thus, in order to reduce the toxicity of LA, we added fish or colza oil to change the FA composition and fluidity of the cell membrane. Our results showed that the viable cell count (Figure 5A) increased after adding fish or colza oil to the medium. One of the main features of PUFA-producing strains are their high level of ROS, which can attack the double bonds of PUFAs and change membrane fluidity. We therefore used exogenous fish or colza oil as ROS targets to mitigate the toxic effect of LA. At the same time, the addition of fish or colza oil caused obvious cytotoxicity in the control strain (Figure 5A) due to the influence of intracellular PUFA. Vitamin C is another pathway that alleviates intracellular ROS toxicity, due to the overexpression of desaturase, when treated with antioxidant vitamin C, ROS and lipid peroxidation were greatly reduced in the fungus *Mucor rouxii* with expressing delta 6 and 12 desaturase, ultimately doubling the lifespan of the PUFA strain [14]. With reference to this paper, similar experimental results were shown in this study.

In conclusion, this study demonstrated that a high content of intrinsically produced LA induces morphological changes and cell death in *Y. lipolytica*. This effect was accompanied by increased ROS and MDA levels, as well as decreases in the cellular activities of the antioxidant enzymes CAT and SOD, indicating that the multicopy strain 1292-12 is affected by increased oxidative stress. These results indicate that the lipotoxicity of excess LA is mediated by oxidative stress, confirming that the fatty acid composition of cellular membranes plays a very important role in many cellular processes. The increase of cell viability and lipid content following the addition of fish or colza oil to the medium confirmed that this simple method is effective for reducing oxidative stress. However, although adding fish or colza to the medium had positive effects on the cell viability and lipid accumulation, the effect was not strong enough to significantly mitigate the lipotoxicity of PUFAs, and further studies are needed to develop more robust PUFAs overproduction strains.

## Figures and Tables

**Table 1:** TFA content and fatty acid profiles of transformants and control strains.

Time (h)	Strains	Fatty Acid Profile (%)					TFA (%)
		C16:0	C16:1	C18:0	C18:1	C18:2	
72	Control	18.1 ± 0.1 ^a^	4.0 ± 0.2 ^a^	11.1 ± 0.4 ^b^	58.1 ± 0.1 ^a^	8.7 ± 0.4 ^d^	6.2 ± 0.2 ^a^
	1312-12	18.6 ± 0.3 ^a^	3.7 ± 0.1 ^a^	12.9 ± 0.3 ^b^	50.2 ± 0.2 ^b^	14.6 ± 0.1 ^c^	6.1 ± 0.1 ^a^
	1292-12	19.4 ± 0. 5 ^a^	1.5 ± 0.2 ^c^	2.7 ± 0.2 ^c^	52.1 ± 0.1 ^b^	24.3 ± 0.5 ^b^	6.0 ± 0.5 ^a^
120	Control	17.4 ± 0.1 ^b^	3.3 ± 0.2 ^a^	15.7 ± 0.3 ^a^	56.7 ± 0.4 ^a^	7.0 ± 0.3 ^d^	5.8 ± 0.4 ^a^
	1312-12	19.5 ± 0.5 ^a^	2.7 ± 0.3 ^b^	10.1 ± 0.4 ^b^	51.0 ± 0.3 ^b^	18.7 ± 0.4 ^c^	6.3 ± 0.3 ^a^
	1292-12	21.8 ± 0.2 ^a^	1.3 ± 0.2 ^c^	3.4 ± 0.4 ^c^	42.7 ± 0.4 ^c^	30.8 ± 0.5 ^a^	6.0 ± 0.4 ^a^

Different superscripts within columns indicate significant (*p* < 0.05) differences between groups.

**Table 2:** Fatty acid profiles of the 1292-12 and control strains when fish or colza oil was added to the medium.

Medium	Time	Strains	Fatty Acid Profile (%)									TFA (%)
			C16:0	C16:1	C18:0	C18:1	C18:2	C18:3	C20:5	C22:1	C22:6	
Added fish oil	72 h	Control	16.6 ± 0.3 ^b^	5.3 ± 0.1 ^a^	9.3 ± 0.2 ^a^	45.0 ± 0.3 ^b^	16.7 ± 0.1 ^b^	-	1.4 ± 0.1 ^b^	-	6.9 ± 0.1 ^a^	6.4 ± 0.2 ^a^
		1292-12	23.5 ± 0.3 ^a^	1.3 ± 0.1 ^d^	2.1 ± 0.1 ^b^	42.4 ± 0.3 ^b^	23.9 ± 0.1 ^a^	-	1.6 ± 0.1 ^b^	-	3.5 ± 0.1 ^b^	6.7 ± 0.6 ^a^
	120 h	Control	20.4 ± 0.2 ^a^	6.5 ± 0.1 ^a^	10.3 ± 0.1 ^a^	40.6 ± 0.3 ^b^	12.3 ± 0.1 ^b^	-	2.6 ± 0.1 ^a^	-	7.5 ± 0.1 ^a^	6.0 ± 0.1 ^a^
		1292-12	20.7 ± 0.1 ^a^	3.7 ± 0.1 ^b^	2.1 ± 0.1 ^b^	45.9 ± 2.3 ^b^	20.4 ± 0.2 ^a^	-	2.1 ± 0.1 ^a^	-	5.2 ± 0.1 ^b^	6.1 ± 0.1 ^a^
Added colza oil	72 h	Control	14.8 ± 0.1 ^c^	2.0 ± 0.1 ^c^	9.5 ± 0.1 ^a^	54.5 ± 0.4 ^a^	14.0 ± 0.1 ^b^	2.5 ± 0.1 ^a^	-	2.8 ± 0.1 ^b^	-	6.8 ± 0.3 ^a^
		1292-12	16.7 ± 0.1 ^b^	2.4 ± 0.1 ^c^	1.9 ± 0.1 ^b^	52.1 ± 0.5 ^a^	22.0 ± 0.3 ^a^	1.0 ± 0.1 ^b^	-	4.1 ± 0.1 ^a^	-	6.0 ± 0.1 ^a^
	120 h	Control	12.6 ± 0.2 ^c^	2.1 ± 0.1 ^c^	8.4 ± 0.1 ^a^	56.7 ± 0.4 ^a^	15.3 ± 0.2 ^b^	2.3 ± 0.1 ^a^	-	2.7 ± 0.1 ^b^	-	6.3 ± 0.1 ^a^
		1292-12	16.3 ± 0.3 ^b^	1.8 ± 0.1 ^c^	2.1 ± 0.1 ^b^	51.6 ± 0.6 ^a^	23.5 ± 0.3 ^a^	2.4 ± 0.1 ^a^	-	2.3 ± 0.1 ^b^	-	6.8 ± 0.1 ^a^

Different superscripts within columns indicate significant (*p* < 0.05) differences between groups.

## Data Availability

Data are contained within the article.
